# Rheumatological features of Whipple disease

**DOI:** 10.1038/s41598-021-91671-9

**Published:** 2021-06-10

**Authors:** Alice Tison, Pauline Preuss, Clémentine Leleu, François Robin, Adrien Le Pluart, Justine Vix, Guillaume Le Mélédo, Philippe Goupille, Elisabeth Gervais, Grégoire Cormier, Jean-David Albert, Aleth Perdriger, Béatrice Bouvard, Jean-Marie Berthelot, Nathan Foulquier, Alain Saraux

**Affiliations:** 1 UMR1227, Lymphocytes B et Autoimmunité, Université de Brest, INSERM, CHU Brest, LabEx IGO, Brest, France; 2 Rheumatology Unit, Centre National de Référence des Maladies Auto-Immunes Rares (CERAINO), CHU, Brest, France; 3 Rheumatology Unit, CHU, Nantes, France; 4 Rheumatology Unit, CHU, Angers, France; 5 Rennes, Service de Rhumatologie, Univ Rennes, INSERM, INRA, Institut NUMECAN (Nutrition Metabolisms and Cancer), 35000 Rennes, France; 6 Rheumatology Unit, CHU, Poitiers, France; 7 Rheumatology Department, University Hospital of Tours, EA 7501, GICC, University of Tours, Tours, France; 8 Rheumatology Unit, CHU, La Roche-sur-Yon, France; 9 Rheumatology Unit, Hôpital de la Cavale Blanche, BP 824, 29609 Brest cedex, France

**Keywords:** Pathogenesis, Rheumatology, Signs and symptoms

## Abstract

Whipple disease (WD) is a rare infectious systemic disease. Rheumatologists are at the frontline of WD diagnosis due to the early rheumatological manifestations. An early diagnosis is crucial, as usual anti-rheumatic drugs, especially TNF inhibitors, may worsen the disease course. We conducted a retrospective multicentre national study from January 2010 to April 2020 to better characterize the rheumatological features of WD. Classic WD (CWD) was defined by positive periodic acid-Schiff (PAS) staining of a small-bowel biopsy sample, and non-CWD (NCWD) was defined by negative PAS staining of a small-bowel biopsy sample but at least one positive Tropheryma whipplei (TW) polymerase chain reaction (PCR) for a digestive or extradigestive specimen. Sixty-eight patients were enrolled, including 11 CWD patients. Twenty patients (30%) received TNF inhibitors during the WD course, with inefficacy or symptom worsening. More digestive symptoms and systemic biological features were observed in CWD patients than in NCWD patients, but both patient groups had similar outcomes, especially concerning the response to antibiotics and relapse rate. Stool and saliva TW PCR sensitivity were both 100% for CWD and 75% for NCWD and 89% and 60% for small-bowel biopsy sample PCR, respectively. WD encountered in rheumatology units has many presentations, which might result from different pathophysiologies that are dependent on host immunity. Given the heterogeneous presentations and the presence of chronic carriage, multiple TW PCR tests on samples from specific rheumatological sites when possible should be performed, but samples from nonspecific digestive and extradigestive sites also have great value.

## Introduction

Described by George Hoyt Whipple in 1907, Whipple disease (WD) is a rare systemic disease caused by a gram-positive intracellular bacterium, *Tropheryma whipplei* (TW). The identification of TW was made possible in the 1990s by the sequencing of its 16S ribosomal RNA^1,2^. Stable culture of the bacteria was obtained in 2000, and the sequencing of the *TW* genome was performed in 2003^3,4^. The annual incidence of WD has been estimated to be less than 1 per 1,000,000 population, and WD preferentially affects middle-aged white men^5^. Classic Whipple disease (CWD), the most documented type of WD, is characterized by the association of nonspecific gastrointestinal symptoms, such as abdominal pain, chronic diarrhoea and weight loss, with articular symptoms, which usually precede the digestive symptoms by several years. Other systemic features may coexist, such as general symptoms (fever, asthenia), cardiac involvement with the occurrence of culture-negative endocarditis, pleural effusion or pericarditis, and sometimes uveitis or a host of nonspecific neurological symptoms (from headache and memory loss to encephalitis and pathognomonic oculomasticatory myorhythmia)^6^. The gold standard for CWD diagnosis is periodic acid-Schiff (PAS)-stained inclusions in foamy macrophages of the lamina propria on small-bowel (SB) biopsy, with possible confirmation by immunohistochemistry using specific anti-TW antibodies^7^. The improvement of polymerase chain reaction (PCR) techniques has enabled the description of localized forms without any digestive involvement based on PCR analysis of specific nondigestive specimens. Some of these forms of WD have been known for years, such as localized culture-negative endocarditis or localized neurological forms. Localized Whipple arthritis (LWA) is increasingly described in the literature^6,8^. TW acute self-limited infections have also been described (gastroenteritis and pneumonitis). Chronic carriage is common among sewer workers due to the faecal-oral transmission of the bacteria^9^. TW has been detected in stool samples in 1 to 11% of healthy individuals and in 12 to 26% of sewage plant workers, and TW has been detected in saliva samples in 0.2% of healthy individuals and in 2.2% of sewage plant workers. In a series of 4418 samples, Fenollar et al. reported a false-positive rate of 2.3% in the detection of TW by PCR for stool samples, 0.2% for saliva samples, and 0.9% for both stool and saliva samples^10^. This carriage reaches 31% in stool samples of healthy individuals in Senegal^11^.

A better understanding of the rheumatological presentation of the disease is necessary to make earlier diagnoses since immunosuppressive (IS) treatments, such as TNF inhibitors (TNFis), may worsen the disease course and may increase the frequency of immune reconstitution inflammatory syndrome (IRIS)^6,12^.

We conducted a retrospective multicentric observational study to better characterize the rheumatological manifestations of WD.

## Methods

### Literature review

A systematic literature review was performed using the artificial intelligence software BIBOT, as previously described^13^, using the keywords detailed in Supplementary Table S1 which included “*Tropheryma whipplei”, “*Clinic” and “Classical—Classic Whipple disease”.

### Study design

We conducted a retrospective observational study through 7 western French centres: Brest, Angers, Rennes, Tours, Nantes, Poitiers, and La Roche-sur-Yon. Patients with a first diagnosis of WD or a late relapse diagnosis made in the rheumatology department between 1 January 2010, and 30 April 2020, were included.

### Microbiology

Diagnosis was made by PAS staining of SB biopsy samples and by real-time quantitative PCR (qPCR) targeting *TW* sequences in various samples*.* A portion of the PCR analyses was performed at the bacteriology laboratory of Marseille Teaching Hospital, which is the French reference centre for WD. The molecular detection of the *TW* genome was performed as described previously^10^. Since 2016, *TW* real-time semiquantitative PCR has been performed at Nantes Teaching Hospital for Nantes, Angers and La Roche-sur-Yon. After extraction, amplification was performed with an amplification kit, TRWH-U (Progenie Molecular), commercialized by Orgentec.

### Diagnosis of WD

All diagnoses were based on 1—rheumatologist expert diagnosis, according to clinical, biological, molecular and histological features; and 2—a spectacular response to antibiotics, including the healing of arthritis and the normalization of CRP levels.

### Subgroups of WD

Patients were divided into the following 4 groups according to histological and molecular findings that enabled the diagnosis:

1. CWD was defined by positive PAS staining of SB biopsy samples (duodenal, jejunal) and sometimes gastric biopsy samples (Group 1).
2. Non-CWD (NCWD) was defined by negative PAS staining or missing data for PAS staining of SB biopsy samples but at least one positive PCR for a digestive or extradigestive specimen. Among patients with NCWD, 3 groups were considered:
  - Localized Whipple disease (LWD) was defined by positive PCR for an extradigestive specific specimen (articular site or cerebrospinal fluid (CSF)). LWA was defined by positive PCR for an articular site specimen (synovial fluid and/or synovial biopsy specimen or discovertebral biopsy specimen) (Group 2).
  - Arthropathic Whipple disease (AWD) was defined by negative PAS staining of SB biopsy samples, negative or no PCR for an extradigestive specific specimen but positive PCR for one or several digestive or nonspecific extradigestive specimens (Group 3).
  - Probably Arthropathic Whipple Disease (PAWD) was defined by missing data for SB biopsy samples or PAS staining and a negative or no PCR for an extradigestive specific specimen but a positive PCR for one or several digestive or nonspecific extradigestive specimens (Group 4).

### Collected data

Demographic characteristics and information on clinical, biological, imaging, molecular and histological features were collected from specific questionnaires in medical records, as well as information on the antibiotics used and the response and evolution of the disease after treatment. The duration of antibiotics was determined from the start of treatment to either the end of treatment or the date of last follow-up. The duration of the follow-up was defined from the date of WD diagnosis to the date of last follow-up.

All methods were carried out in accordance with relevant guidelines and regulations and all protocols were approved by a named institutional and/or licensing committee/s: This study was approved by the French National Data Protection Commission and by the Brest University Hospital ethics committee (#2017CE.19/1) and declared a clinical trial (NCT03350685). Informed consent was obtained from all subjects.

### Statistical analysis

Chi-squared or Fisher tests were performed to compare categorical data between groups, and Mann–Whitney tests were used to compare continuous variables. SPSS software version 25.0 [IBM, Armonk, NY, USA] was used for all analyses. All significance tests were two-tailed, and *p* values < 0.05 were considered significant.

## Results

### Literature review

A total of 206 articles published from 1995 to July 2020 were obtained. Searches with specific keywords were then performed on articles’ MESH terms, keywords and abstracts to select the articles that compared CWD and LWA. Articles dealing only with CWD or extrarheumatological LWD were excluded. Finally, 26 articles^10,14–20^ were retained for analysis, including 10 series, 14 reviews and 2 case reports. Eight series reported outcomes for patients with CWD versus LWA. They are summarized in Supplementary Table S2.

### Patients’ general characteristics

Sixty-eight patients were included between January 2010 and April 2020. The general characteristics and clinical and laboratory features of the patients according to WD presentation at diagnosis are presented in Table 1. Eleven patients were diagnosed with CWD, 24 patients with LWD, and 22 patients with LWA. Fifteen patients were diagnosed with AWD, and 18 patients were diagnosed with PAWD. Two patients worked in contact with wastes/sewer. Most patients (72%) received IS treatment during their disease course. Among the 20 patients treated with TNFis, inefficacy was described in 7 and a worsening of symptoms was described in 9. Three patients treated with TNFis experienced an improvement in symptoms (with secondary inefficacy or partial improvement).

**Table 1:** Population general characteristics and clinical and laboratory features according to Whipple disease entity.

	CWD N = 11	LWD N = 24	AWD N = 15	PAWD N = 18	Total N = 68
**General characteristics n/N (%)**					
Males	9/11 (82)	21/24 (88)	13/15 (87)	13/18 (72)	56/68 (82)
Median age at diagnosis, years [range]	60 [34–79]	63 [42–79]	61 [42–77]	53 [30–80]	60 [30–80]
Familial history of autoimmunity	3/8 (38)	3/21 (14)	3/10 (30)	7/15 (47)	16/54 (30)
First-degree familial history of Whipple disease	1/8 (13)	2/20 (10)	0/10 (0)	1/16 (6.3)	4/54 (7.4)
Median duration before diagnosis, years [range]	7 [0–16]	5 [1–31]	4 [0–20]	3 [0–40]	5 [0–40]
Alternative diagnosis	7/9 (78)	17/21 (81)	14/14 (100)	13/15 (87)	51/59 (86)
**Clinical features n/N (%)**					
Abdominal pain	3/11 (27)	3/24 (13)	3/15 (20)	2/18 (11)	11/68 (16)
Weight loss	10/11 (91)	5/23 (22)	8/14 (57)	6/17 (35)	29/65 (45)
Diarrhoea	6/11 (55)	7/24 (29)	3/15 (20)	4/18 (22)	20/68 (29)
Arthritis	10/11 (91)	22/24 (92)	13/15 (87)	15/18 (83)	60/68 (88)
Inflammatory arthralgia	11/11 (100)	21/24 (88)	12/15 (80)	18/18 (100)	62/68 (91)
Inflammatory back pain	5/11 (45)	7/23 (30)	6/15 (40)	7/18 (39)	25/67 (37)
Fever	5/11 (45)	4/23 (17)	3/15 (20)	4/18 (22)	16/67 (24)
Adenopathy	4/10 (40)	3/22 (14)	7/15 (47)	1/18 (5.6)	15/65 (23)
Pleural effusion	1/11 (9.1)	0/24 (0)	0/15 (0)	1/18 (5.6)	2/68 (2.9)
Pericarditis	0/11 (0)	1/24 (4.2)	4/15 (27)	1/18 (5.6)	6/68 (8.8)
Endocarditis	1/11 (9.1)	1/24 (4.2)	0/15 (0)	1/18 (5.6)	3/68 (4.4)
Neurological symptoms	0/11 (0)	1/24 (4.2)	2/15 (13)	1/18 (5.6)	4/68 (5.9)
Uveitis	1/11 (9.1)	1/24 (4.2)	0/15 (0)	0/18 (0)	2/68 (2.9)
Melanoderma	0/11 (0)	2/24 (8.3)	2/15 (13)	0/18 (0)	4/68 (5.9)
**Biological features n/N (%)**					
Elevated CRP	10/11 (91)	21/24 (88)	15/15 (100)	17/18 (94)	63/68 (93)
Median CRP, mg/l [range]	80 [52–237]	51 [9–235]	64 [21–150]	55 [11–222]	58.5 [9–237]
Median albuminemia, g/l [range]	29 [23–35]	38 [21–45]	36 [22–41]	38 [27–43]	36 [21–45]
Anaemia	9/11 (82)	6/24 (25)	7/14 (50)	5/17 (29)	27/66 (41)
Eosinophilia	0/10 (0)	1/24 (4.2)	1/14 (7.1)	0/17 (0)	2/65 (3.1)
Thrombocytosis	6/11 (55)	4/24 (17)	3/13 (23)	4/17 (24)	17/65 (26)
Neutrophilia	4/11 (36)	9/24 (38)	8/14 (57)	6/18 (33)	27/67 (40)
Polyclonal hypergammaglobulinemia	3/8 (38)	1/22 (4.5)	3/13 (23)	1/15 (6.7)	8/58 (14)
Serum hyperIgA > 3.5 g/l	2/4 (50)	1/5 (20)	5/6 (83)	2/5 (40)	10/20 (50)
Elevated CSF proteins	0/1 (0)	4/8 (50)	3/5 (60)	3/7 (43)	10/21 (48)
RF	0/11 (0)	1/20 (5)	3/13 (23)	1/17 (5.9)	5/61 (8.2)
Anti-CCP	0/11 (0)	0/20 (0)	0/13 (0)	0/15 (0)	0/59 (0)
HLA B27	2/5 (40)	3/8 (38)	0/7 (0)	3/9 (33)	8/29 (28)
ANA ≥ 1/160	2/11 (18)	6/20 (30)	5/14 (36)	4/17 (24)	17/62 (27)
ANCA	1/6 (17)	0/9 (0)	1/7 (14)	0/10 (0)	2/32 (6.3)
Immunosuppressive treatment n/N (%)	8/11 (73)	15/24 (63)	12/15 (80)	14/18 (78)	49/68 (72)
Glucocorticoids	8/11 (73)	11/20 (55)	10/13 (77)	9/16 (56)	38/60 (63)
csDMARD *	8/11 (73)	12/24 (50)	11/15 (73)	11/17 (65)	42/67 (63)
Inefficacy	4/8 (50)	9/10 (90)	8/11 (73)	10/11 (91)	31/40 (78)
Symptom improvement	2/8 (25)	1/10 (10)	3/11 (27)	1/11 (9.1)	7/40 (18)
Symptom worsening	2/8 (25)	0/10 (0)	0/11 (0)	0/11 (0)	2/40 (5)
Biologic**	5/11 (45)	3/24 (13)	7/15 (47)	5/17 (29)	20/67 (30)
Inefficacy	1/5 (20)	2/3 (67)	2/6 (33)	2/5 (40)	7/19 (37)
Symptom improvement	1/5 (20)	0/3 (0)	2/6 (33)	0/5 (0)	3/19 (16)
Symptom worsening	3/5 (60)	0/3 (0)	1/6 (17)	1/5 (20)	5/19 (26)
Improvement with secondary worsening	0/5 (0)	1/3 (33)	1/6 (17)	2/5 (40)	4/19 (21)
Median follow-up, m [range]	68 [13–189]	24 [1–82]	44 [16–101]	35 [9–120]	33 [1–189]

*ANA* antinuclear antibodies; *AWD* arthropathic Whipple disease; *CSF* cerebrospinal fluid; *CWD* classic Whipple disease; *csDMARD* conventional synthetic disease-modifying anti-rheumatic drug; *LWD* localized Whipple disease; *PAWD* probably arthropathic Whipple disease; *RF* rheumatoid factor.

*Methotrexate, salazopyrin, leflunomide, hydroxychloroquine, gold, azathioprine.

**TNF inhibitor, abatacept, IL 12/23 inhibitor, IL 17 inhibitor, rituximab, IL1 inhibitor.

The number of diagnoses of the forms of WD among the 6 French centres is presented in Supplementary Table S3. The evolution of WD diagnoses across years is presented in Supplementary Fig. S1.

### Clinical features

All patients but one presented rheumatological symptoms. Specific rheumatological features are presented in Table 2. Forty-two patients (62%) exclusively had peripheral involvement, while 26 patients (38%) had axial + /− peripheral involvement. Arthritis occurred in 60 patients (88%), inflammatory arthralgia occurred in 62 patients (91%), and inflammatory back pain occurred in 25 patients (37%). The more involved joints were the knee, ankle and wrist (Supplementary Table S4). Oligoarticular and polyarticular involvement were seen in 48% and 44% of our population, respectively. Palindromic rheumatism was observed in 42 patients, with a trend towards more intermittent presentation in LWD (90%), AWD (92%) and PAWD (67%) than in CWD (50%). Three patients presented enthesitis associated to arthritis. Twenty-two patients (32%) had digestive involvement (abdominal pain and/or diarrhoea). Twenty-nine patients presented with weight loss, 16 with fever and 15 with adenopathy. Three patients presented with endocarditis. Neurological involvement was rare (4 patients with nonspecific symptoms such as visual hallucinations, memory loss, headache, erectile dysfunction). One of these patients had a positive CSF PCR. Four patients had melanoderma, and 3 had extensive nodular eruptions on the front of the limbs (one erythema nodosum).

**Table 2:** Rheumatological clinical and imaging features according to Whipple disease entity.

	CWD N = 11	LWD N = 24	AWD N = 15	PAWD N = 18	Total N = 68
**Clinical features n/N (%)**					
Exclusive peripheral involvement	6/11 (55)	15/24 (63)	9/15 (60)	12/18 (67)	42/68 (62)
Axial +/− peripheral involvement	5/11 (45)	9/24 (38)	6/15 (40)	6/18 (33)	26/68 (38)
Arthritis	10/11 (91)	22/24 (92)	13/15 (87)	15/18 (83)	60/68 (88)
Inflammatory arthralgia	11/11 (100)	21/24 (88)	12/15 (80)	18/18 (100)	62/68 (91)
Monoarticular involvement	1/7 (14)	2/22 (9.1)	0/10 (0)	1/13 (7.7)	4/52 (7.7)
Oligoarticular involvement	3/7 (43)	12/22 (55)	3/10 (30)	7/13 (54)	25/52 (48)
Polyarticular involvement	3/7 (43)	8/22 (36)	7/10 (70)	5/13 (38)	23/52 (44)
Symmetric	3/8 (38)	8/20 (40)	4/8 (50)	5/9 (56)	20/45 (44)
Intermittent	4/8 (50)	19/21 (90)	11/12 (92)	8/12 (67)	42/53 (79)
Inflammatory back pain	5/11 (45)	8/24 (33)	5/14 (36)	7/18 (39)	25/67 (37)
**Imaging features n/N (%)**					
Radiographic joint space narrowing	4/7 (57)	4/13 (31)	4/8 (50)	4/13 (31)	16/41 (39)
Radiographic erosions	4/10 (40)	4/21 (19)	1/10 (10)	1/15 (6.7)	10/56 (18)
Radiographic destruction	2/10 (20)	2/19 (11)	2/10 (20)	0/14 (0)	6/53 (11)
Spondylodiscitis on MRI	0/0 (0)	2/8 (25)	1/3 (33)	0/2 (0)	3/13 (23)
Sacroiliitis on CT and/or MRI	2/3 (67)	1/5 (20)	3/4 (75)	1/3 (33)	7/15 (47)

*AWD* arthropathic Whipple disease; *CT* computed tomography; *CWD* classic Whipple disease; *LWD* localized Whipple disease; *MRI* magnetic resonance imaging; *PAWD* probably arthropathic Whipple disease.

### Laboratory features

Table 1 shows biological data. Sixty-three patients had elevated CRP levels. Among those with normal CRP levels, 1 had CWD, 3 had LWD and 1 had PAWD. Anaemia and neutrophilia were each observed in 27 patients. Eosinophilia was rare. Eight patients had polyclonal hypergammaglobulinemia, and 10 patients had an increase in serum IgA > 3.5 g/l (4 had both hypergammaglobulinemia and hyperIgA, including one with IgA nephropathy and one with monoclonal IgA lambda gammopathy). Five patients had rheumatoid factor. No patients had anti-CCP antibodies. HLA B27 was positive in 8/29 patients (28%) tested.

### Imaging features

Radiographic erosions were seen in 10 patients (18%) (Table 2). There was no difference in erosion occurrence according to whether TNFis were used or according to whether CRP was ≥ 50 mg/l or < 50 mg/l. Sacroiliitis on computed tomography or magnetic resonance imaging was observed in 7 patients, while 3 presented spondylodiscitis.

### Diagnostic methods

The histological and molecular methods used for WD diagnosis are presented in Table 3. By definition, 100% of patients with CWD had positive PAS staining of SB biopsy samples, whereas no patients with NCWD did. Saliva and stool PCR sensitivity were both 100% for CWD and 75% for NCWD. SB biopsy sample PCR sensitivity was 89% for CWD versus 60% for NCWD. Urine and blood PCR were less sensitive (33% and 50% versus 13% and 23% for CWD versus NCWD, respectively). Among patients with NCWD, all but 2 had at least two positive digestive or extradigestive specimen PCRs.

**Table 3:** Diagnostic methods according to Whipple disease entity.

	CWD N = 11	LWD N = 24	AWD N = 15	PAWD N = 18	Total N = 68
PAS staining n/N (%)	11/11 (100)	0/12 (0)	0/15 (0)	ND	
**PCR n/N (%)**					
Cutaneous biopsy	2/2 (100)	0/2 (0)	0/1 (0)	3/8 (38)	5/13 (38)
Saliva	10/11 (91)	15/21 (71)	13/16 (81)	14/18 (78)	52/66 (79)
Stool	9/9 (100)	21/22 (95)	14/14 (100)	17/18 (94)	61/63 (97)
Saliva and stool	9/9 (100)	15/21 (71)	12/14 (86)	13/18 (72)	49/62 (79)
Small-bowel biopsy	8/9 (89)	7/17 (41)	8/14 (57)	10/11 (91)	33/51 (65)
Synovial fluid	3/5 (60)	21/21 (100)	0/1 (0)	0/1 (0)	24/28 (86)
Synovial biopsy	1/1 (100)	1/2 (50)	0/1 (0)	0/1 (0)	2/5 (40)
Discovertebral biopsy	0	1/2 (50)	0/1 (0)	0	1/3 (33)
CSF	1/6 (17)	3/11 (27)	0/5 (0)	0/9 (0)	4/31 (13)
Cardiac valve	1/1 (100)	0	0	0	1/1 (100)
Urine	1/3 (33)	0/3 (0)	1/2 (50)	0/3 (0)	2/11 (18)
Blood	5/10 (50)	2/9 (22)	2/4 (50)	1/9 (11)	10/32 (31)

*AWD* arthropathic Whipple disease; *CSF* cerebrospinal fluid; *CWD* classic Whipple disease; *LWD* localized Whipple disease; *PAS* periodic acid-Schiff; *PAWD* probably athropathic Whipple disease.

### Alternative diagnoses

Fifty-one patients had an alternative diagnosis before the diagnosis of WD. According to rheumatological features, spondyloarthritis, psoriatic arthritis (PsA) and rheumatoid arthritis (RA) were initially diagnosed in 11, 7 and 9 patients, respectively. Unclassified rheumatism was diagnosed in 10 patients, with a palindromic form for 5/7 patients, while a microcrystal aetiology was suspected in 5 patients. One patient had a diagnosis of connective tissue disease, and 2 had a diagnosis of vasculitis. A diagnosis of sarcoidosis was suspected in three patients.

### Antibiotic use, response and evolution post treatment

Most antibiotic treatments consisted of a combination of doxycycline and hydroxychloroquine (n = 63). Two patients received doxycycline alone. Seven patients received trimethoprim sulfamethoxazole. The mean duration of antibiotic treatment was 20 months (P25%0–P75%75) (data available for 35 patients).

The disease evolution after antibiotic treatment is presented in Supplementary Table S5. None of the 68 patients had resistance to antibiotic treatment, with a rapid relief of symptoms and inflammatory syndrome resolution. At the end of antibiotic treatment, 36/43 tested patients had negative PCR results (84%). IRIS was rare in the cohort (5 patients). Relapses occurred in 21/65 patients (32%).

Seven patients in the cohort evolved towards chronic rheumatism, all at least one year after antibiotic treatment initiation: 2 with CWD, 1 with LWA and 4 with AWD/PAWD. The characteristics of these patients are presented in Supplementary Table S6.

### Evolutive complications

Two diagnoses of lymphoma during the disease course and 4 diagnoses of monoclonal gammopathy of undetermined significance (MGUS) were reported in NCWD patients. One patient was diagnosed with extranodal diffuse large B cell lymphoma (DLBCL) 4 years after WD diagnosis. Another patient was diagnosed with marginal zone lymphoma and Hodgkin lymphoma (HL) the year of WD diagnosis, 1 year after the beginning of symptoms. Two patients in the cohort had a history of spontaneous deep vein thrombosis (DVT) during the disease course. One patient had CWD and the other one had AWD. Both patients had CRP > 100 mg/l at WD diagnosis and low albumin (29 and 22 g/l). Of note, the first patient was diagnosed with polygcythemia 5 years later.

### Comparison of CWD versus NCWD

General, clinical, biological, imaging and therapeutic features according to CWD versus NCWD are presented in Table 4. Overall, in our cohort, patients with CWD and NCWD had similar features. Patients with CWD had more digestive symptoms (abdominal pain, diarrhoea and weight loss, p = 0.247, p = 0.046 and p = 0.001, respectively). The rheumatological feature frequency was similar between groups, except for the proportion of patients with palindromic presentation being higher among NCWD patients (p = 0.048). Systemic biological signs were more pronounced in CWD patients, with higher CRP levels, lower albuminemia levels, and more anaemia and thrombocytosis. IRIS and relapse rates did not differ among groups.

**Table 4:** General, clinical, biological, imaging and therapeutic features according to classic versus non classic Whipple disease.

	CWD N = 11	NCWD N = 57	P value
**General characteristics n/N (%)**			
Male	9/11 (82)	47/57 (82)	0.624
Median age at diagnosis, years [range]	60 [34–79]	60 [30–80]	0.764
Familial history of autoimmunity	3/8 (38)	13/46 (28)	0.441
First-degree familial history of Whipple disease	1/8 (13)	3/46 (6.5)	0.484
Median duration before diagnosis, years [range]	7 [0–16]	4 [0–40]	0.640
Alternative diagnosis	7/9 (78)	44/50 (88)	0.352
**Clinical features n/N (%)**			
Abdominal pain	3/11 (27)	8/57 (14)	**0.247**
Weight loss	10/11 (91)	19/54 (35)	**0.001**
Diarrhoea	6/11 (55)	14/57 (25)	**0.046**
Arthritis	10/11 (91)	50/57 (88)	0.617
Hip arthritis	2/11 (18)	1/57 (1.8)	0.066
Inflammatory arthralgia	11/11 (100)	51/57 (89)	0.332
Intermittent	4/8 (50)	38/45 (84)	**0.048**
Axial +/− peripheral involvement	6/11 (55)	36/57 (63)	0.590
Fever	5/11 (45)	11/56 (20)	0.066
Adenopathy	4/10 (40)	11/55 (20)	0.164
Pleural effusion	1/11 (9.1)	1/57 (1.8)	0.299
Pericarditis	0/11 (0)	6/37 (16)	0.332
Endocarditis	1/11 (9.1)	2/57 (3.5)	0.416
Neurological symptoms	0/11 (0)	4/57 (7.0)	0.485
Uveitis	1/11 (9.1)	1/57 (1.8)	0.299
Melanoderma	0/11 (0)	4/57 (7.0)	0.485
**Biological features n/N (%)**			
Elevated CRP	10/11 (91)	53/57 (93)	0.598
Median CRP mg/l[range]	80 [52–237]	56 [9–235]	**0.016**
Median albuminemia g/l [range]	29 [23–35]	38 [21–45]	**0.001**
Anaemia	9/11 (82)	18/55 (33)	**0.004**
Thrombocytosis	6/11 (55)	11/54 (20)	**0.019**
Polynucleosis	4/11 (36)	23/56 (41)	0.524
RF	0/11 (0)	5/50 (10)	0.356
Anti-CCP	0/11 (0)	0/48 (0)	0
HLA B27	2/5 (40)	6/24 (25)	0.425
ANA > = 1/160	2/11 (18)	15/51 (29)	0.364
ANCA	1/6 (17)	1/26 (3.8)	0.345
**Imaging features n/N (%)**			
Joint space narrowing	4/7 (57)	12/34 (35)	0.254
Radiographic erosions	4/10 (40)	6/46 (13)	0.066
**Immunosuppressive treatment n/N (%)**			
Glucocorticoids	8/11 (73)	30/49 (61)	0.363
csDMARD	8/11 (73)	34/56 (61)	0.347
Biologic	5/11 (45)	15/56 (27)	0.216
**Evolution after antibiotherapy n/N (%)**			
IRIS	2/11 (18)	3/53 (5.7)	0.201
Relapse	5/11 (45)	16/54 (30)	0.306
Evolution towards chronic rheumatism	2/11 (18)	5/53 (9.4)	0.345

*ANA* antinuclear antibodies; *CWD* classic Whipple disease; *csDMARD* conventional synthetic disease-modifying antirheumatic drug; *IRIS* Immune reconstitution inflammatory syndrome; *NCWD* nonclassic Whipple disease; *RF* rheumatoid factor.

## Discussion

In this multicentre rheumatological observational cohort study, we identified 68 patients with WD, all but one with rheumatological involvement, of whom 60 had arthritis, 63 had elevated CRP levels, 20 had ineffective biological therapy, and all had a response to antibiotics. This rheumatological cohort is the largest described so far, with 22 patients with LWA, a presentation with scarce data in the literature. We also propose a new concept of AWD and PAWD for patients with compatible symptoms but negative or unrealized PAS staining of SB biopsy samples but with several positive PCRs for digestive or nonspecific extraintestinal specimens, with a spectacular response to antibiotics and CRP normalization. We compared 11 patients with CWD and 57 patients with NCWD, and overall, we found similar features, especially in terms of response to antibiotic treatment and long-term disease evolution. Nevertheless, more digestive and systemic involvement was found in CWD patients, as well as a trend towards more erosions in this group. Our systematic review found 8 series addressing the same issue, which are summarized in Supplementary Table S2. The overall sex ratio and age were comparable, with a higher percentage of females reported in the LWD group in the series of Lehman et al.^14–17^. The mean disease durations before diagnosis were also comparable^14,15,17^.

As in our cohort, more gastrointestinal symptoms, systemic symptoms and elevated inflammatory markers were reported in CWD patients than in NCWD patients^14,15^. Rheumatological features were classically reported as prodromal symptoms in CWD patients^14–16,18^. When performed, CSF PCR was found to be positive in 25 to 47% of patients, even in the absence of neurological symptoms^15,16,18^. This finding is consistent with the results observed in a cohort of 191 CWD patients, in whom 41% had a positive CSF PCR, with a higher frequency being observed in patients with neurological symptoms^21^. Patients with LWA also reported arthralgia several years before diagnosis^5^. In Crews et al.^15^, half of the patients reported systemic symptoms such as fatigue and night sweats, and elevated inflammatory markers were also increased in nearly half of the patients. Synovial fluid PCR was positive in 85% of patients. The most common rheumatological feature described in the literature was chronic intermittent oligoarthritis but also polyarthritis^17,19^; in our series, the proportions of patients with oligoarthritis and polyarthritis were the same. To summarize, WD should first be suspected in patients with chronic intermittent seronegative arthritis, in middle-aged men, especially patients in whom IS treatment is ineffective and in those with persistent inflammatory syndrome or neutrophilia^8^.

Whether LWA and CWD are independent entities or whether LWA could evolve towards CWD is a matter of debate. A continuum could exist between them, especially when IS treatment is used^6,12^. Nevertheless, in our cohort, we did not find any difference in terms of symptom duration before diagnosis between the CWD group and the NCWD group, and biologic use history was also similar between groups. Nevertheless, WD entities could be independent due to different host immunity. Several strains of TW have been described that do not have different influences on the course of infection^5^. The *TW* genome suggests a host-dependent lifestyle, with the requirement of external nutrients but also the use of mechanisms to escape from the host immune system^6^. WD infection is thought to result from a subtle defect of host immunity, explaining the scarcity of invasive infection compared to the prevalence of chronic carriage and the possibility of reinfections, sometimes with a different strain^22^. Some genetic associations have been described supporting this hypothesis, such as the association with HLA alleles DRB1*13 and DQB1*06^6,23^. Here, we only collected HLA B27. The digestive lumen is the site of TW multiplication, where phagocytosis by macrophages is inefficient to kill the bacteria, leading to an insufficient TH1 response^8,9^. M2 macrophage polarization is observed, along with an IS milieu in the lamina propria and in the serum^6,9,22^. All these elements can lead to invasive infection.

Immune system overactivity linked to antigen stimulation is also seen. Family history of autoimmune diseases is not more common in non classic WD than in classic WD in this cohort, suggesting that autoimmune features are the result of infection with TW in general. Besides, positive ANA was one of the common findings in this cohort, whereas neither RF nor anti-CCP antibody was detected but they are also probably a consequence of infection rather than an autoimmune feature predisposing to WD. Supporting immune system overactivity, a preponderant lymphocyte T (LT)-mediated granuloma formation explains sarcoid-like WD infection with nodular eruptions and polyadenopathy.

The role of B cells has also been reported in WD, with peripheral B cell disturbance^24^. Impaired secretory IgA production has also been described in the gut, along with an increase in the peripheral IgA subclass and a decrease in the IgM and IgG2 subclasses^22^. Chronic B cell activation could explain hypergammaglobulinemia and RF positivity in some patients, with a possible evolution towards clonal expansion and even lymphoma, as observed in two patients in our cohort. This phenomenon has already been observed for MALT lymphoma in other infections, such as *Helicobacter pylori*, or some connective tissue diseases, such as RA or Sjögren syndrome.

Finally, LWA and AWD entities could be the result of T-cell-mediated reactive arthritis. Indeed, Dolmans et al.^9^ hypothesized that extracellular forms of TW bacteria forming aggregates in the extracellular matrix could represent a dormant form of TW in view of its absolute need for intracellular nutrients to survive. Intra-articular niches of this dormant germ could explain negative synovial fluid PCR in some patients with AWD, and reactive arthritis could also be the result of impaired antigen presentation with cross-reactivity between TW and intra-articular antigens. This mechanism could explain the evolution of LWA and AWD towards chronic undefined rheumatism, as reported for 7 patients in our cohort.

Of note, 2 patients in our cohort presented spontaneous DVT, which could be linked to chronic inflammation along with hypoalbuminemia^25,26^.

**Figure 1:**
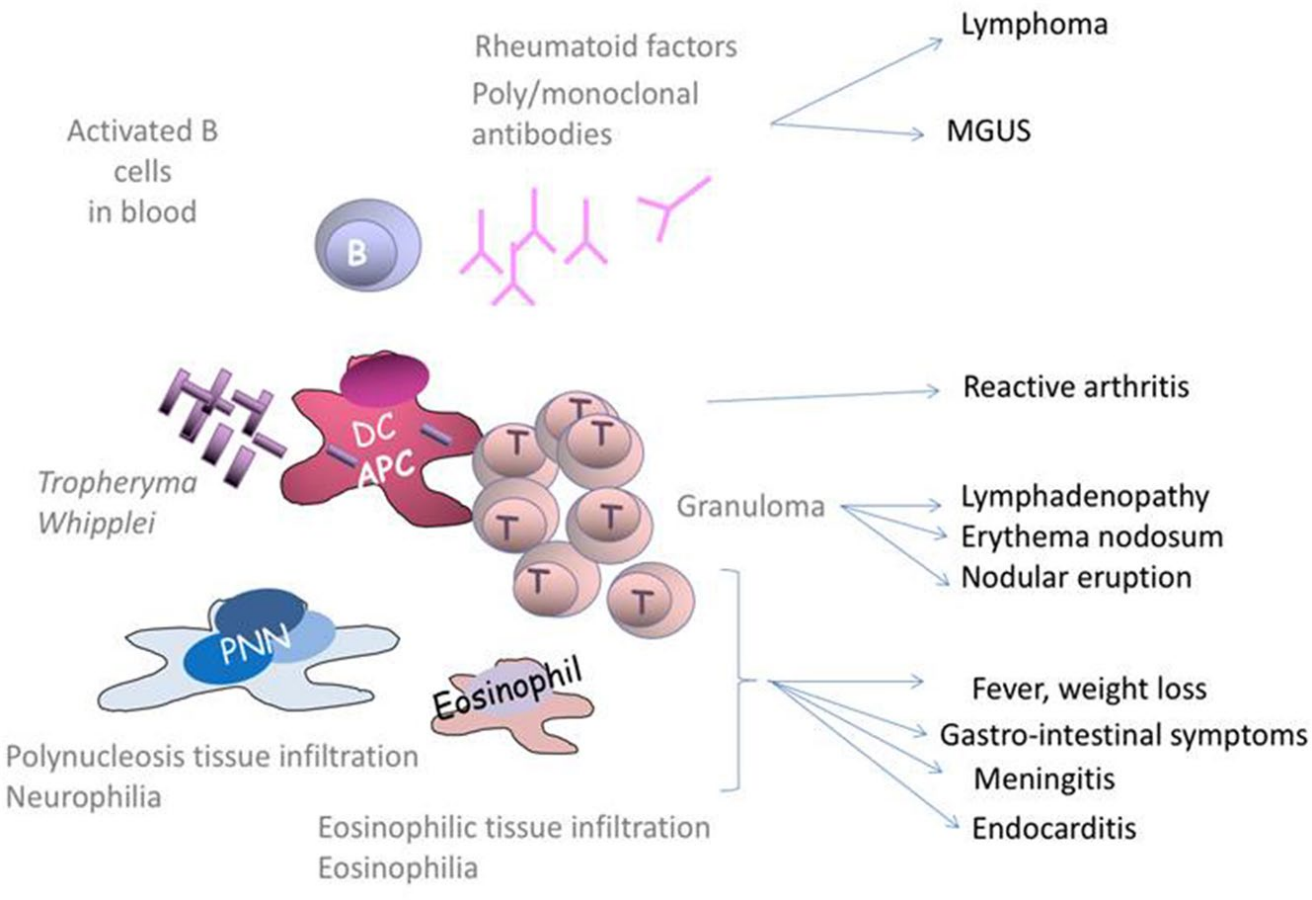
Proposed overactive immune actors involved in Whipple disease pathophysiology. *APC* antigen presentation cell, *DC* dendritic cell, *PNN* neutrophils polynuclears, *T* Lymphocytes T, *WD* Whipple disease.

In summary, WD has many presentations, with an initial immune defect in the gut. Other immune actors are also involved, with global overactivity in response to chronic antigen stimulation, and are responsible for the various WD presentations encountered. The proposed overactive immune actors involved in WD pathophysiology are presented in Fig. 1.

Our study has several limitations. One of them is the scarcity of histological data concerning immunohistochemical analysis of SB biopsy samples. Thus, we might have missed some cases of CWD, when immunohistochemistry is more sensitive than PAS staining^7^. The second limitation is the employment of different PCR methods at the Nantes and Marseille centres and, thus, a possible centre effect. Nevertheless, WD diagnosis rates were comparable between the Brest and Nantes centres. Another limitation is our classification of WD according to histological and molecular features instead of clinical features. The CWD concept is well documented in the literature to be based on histological positive PAS staining +/− positive immunohistochemistry of SB biopsy samples. This technique lacks sensitivity, especially in localized forms. With cases of chronic carriage, misdiagnoses with nonspecific sample PCRs are indeed possible, and in cases of PAS staining negativity, most of the authors advise supplementing with PCR on specific specimens, such as synovial fluid, CSF, and adenopathy, the result of which determines the LWD form. Stool and saliva PCR have demonstrated good sensitivity for the identification CWD with the proposal of new algorithms. Nevertheless, this test combination showed poor performance in patients with LWD, with the limit that LWD in the literature focused on localized endocarditis or neurological forms, with very few data on LWA^8,10^. In LWD patients with positive results for both saliva and stool PCR, Fenollar et al.^10^ reported a sensitivity of 58%, compared with 94% in CWD patients. In our cohort, stool and saliva PCR both had a sensitivity of 100% for CWD and 75% for NCWD diagnosed in rheumatology units. The SB biopsy sample PCR sensitivity fell to 60% for NCWD (89% for CWD). Thus, SB biopsy sample PCR also has a place in the diagnostic algorithm and seems specific^10^. Although specific, urine PCR lacked sensitivity, especially in LWD^10,27,28^.

**Figure 2:**
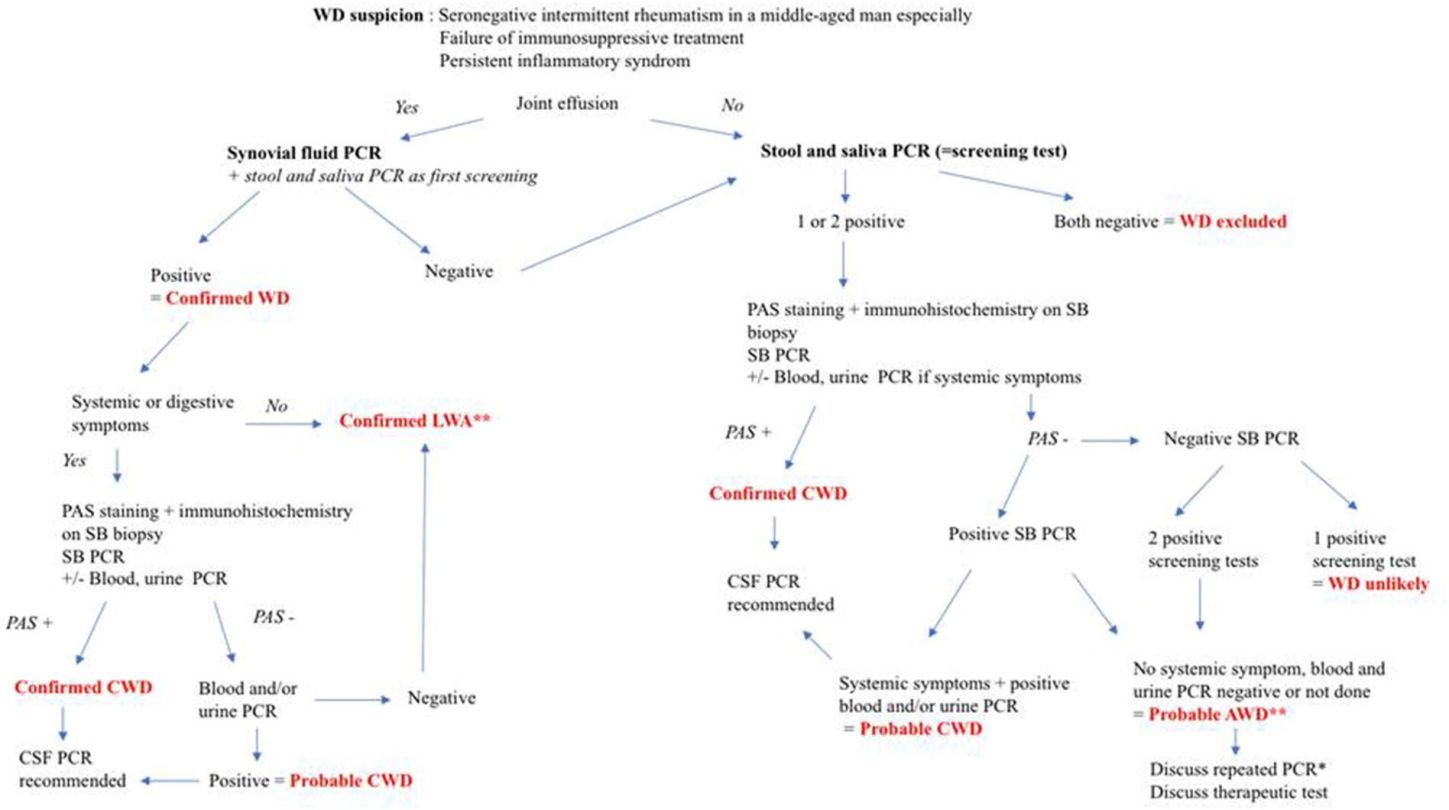
Proposed algorithm for Whipple disease in patients in whom rheumatism is suspected. *CSF* cerebro-spinal fluid, *CWD* classic Whipple disease, *PAS* periodic acid-Schiff, *SB* small bowel, *WD* Whipple disease. Cases of spondylodiscitis not considered in this algorithm. **Discuss CSF PCR in case of neurologic symptoms. *In case of chronic arthritis with negative synovial fluid PCR, discuss synovial biopsy. *NB* Data on cutaneous biopsy PCR were to scarce in our cohort integrate it on the algorithm.

As the rheumatologist is at the frontline for WD diagnosis, with most patients presenting with NCWD, these findings prompt, in patients with suspected rheumatism, an expansion of the panel of TW PCR tests to samples from specific and nonspecific sites. We propose an algorithm for WD diagnosis in rheumatology units (Fig. 2). It is also important to exclude inflammatory bowel diseases based on endoscopic and histopathological findings.

In summary, CWD versus LWA classification should be performed on the basis of clinical and biological features rather than histological and molecular findings. WD encountered in rheumatology units are often nonclassic and seem to be dependent on host immunity, with subtle immunosuppression, at least in the intestinal mucosa, but some entities are suggestive of reactive arthritis with evidence of overactive systemic immunity.

## Supplementary Information

Supplementary Tables.

Supplementary Figure.

## Data Availability

Patients were not involved in this study but they received an information about it.

## References

[1] Black-Schaffer B (1949). The tinctoral demonstration of a glycoprotein in Whipple’s disease. Proc. Soc. Exp. Biol. Med..

[2] Wilson KH, Blitchington R, Frothingham R, Wilson JA (1991). Phylogeny of the Whipple’s-disease-associated bacterium. Lancet.

[3] Raoult D, Birg ML, La Scola B, Fournier PE, Enea M, Lepidi H (2000). Cultivation of the bacillus of Whipple’s disease. N. Engl. J. Med..

[4] Bentley SD, Maiwald M, Murphy LD, Pallen MJ, Yeats CA, Dover LG (2003). Sequencing and analysis of the genome of the Whipple’s disease bacterium Tropheryma whipplei. Lancet.

[5] Schneider T, Moos V, Loddenkemper C, Marth T, Fenollar F, Raoult D (2008). Whipple’s disease: New aspects of pathogenesis and treatment. Lancet Infect. Dis.

[6] Marth T, Moos V, Müller C, Biagi F, Schneider T (2016). Tropheryma whipplei infection and Whipple’s disease. Lancet Infect. Dis..

[7] Baisden BL, Lepidi H, Raoult D, Argani P, Yardley JH, Dumler JS (2002). Diagnosis of Wihipple disease by immunohistochemical analysis: A sensitive and specific method for the detection of Tropheryma whipplei (the Whipple bacillus) in paraffin-embedded tissue. Am. J. Clin. Pathol..

[8] Puéchal X (2013). Whipple’s disease. Ann. Rheum. Dis..

[9] Dolmans RAV, Boel CHE, Lacle MM, Kusters JG (2017). Clinical manifestations, treatment, and diagnosis of tropheryma whipplei infections. Clin. Microbiol. Rev..

[10] Fenollar F, Laouira S, Lepidi H, Rolain J-M, Raoult D (2008). Value of Tropheryma whipplei quantitative polymerase chain reaction assay for the diagnosis of Whipple disease: Usefulness of saliva and stool specimens for first-line screening. Clin. Infect. Dis..

[11] Keita AK, Bassene H, Tall A, Sokhna C, Ratmanov P, Trape J-F (2011). Tropheryma whipplei: A common bacterium in rural Senegal. PLoS Negl Trop Dis..

[12] Marth T (2014). Complicated Whipple’s disease and endocarditis following tumor necrosis factor inhibitors. World J. Cardiol..

[13] Orgeolet L, Foulquier N, Misery L, Redou P, Pers J-O, Devauchelle-Pensec V (2020). Can artificial intelligence replace manual search for systematic literature? Review on cutaneous manifestations in primary Sjögren’s syndrome. Rheumatology (Oxford).

[14] Lehmann P, Ehrenstein B, Hartung W, Dragonas C, Reischl U, Fleck M (2017). PCR analysis is superior to histology for diagnosis of Whipple’s disease mimicking seronegative rheumatic diseases. Scand. J. Rheumatol..

[15] Crews NR, Cawcutt KA, Pritt BS, Patel R, Virk A (2018). Diagnostic approach for classic compared with localized Whipple disease. Open Forum Infect. Dis..

[16] Hujoel IA, Johnson DH, Lebwohl B, Leffler D, Kupfer S, Wu T-T (2019). Tropheryma whipplei Infection (Whipple Disease) in the USA. Dig. Dis. Sci..

[17] Glaser C, Rieg S, Wiech T, Scholz C, Endres D, Stich O (2017). Whipple’s disease mimicking rheumatoid arthritis can cause misdiagnosis and treatment failure. Orphanet J. Rare Dis..

[18] Lagier J-C, Lepidi H, Raoult D, Fenollar F (2010). Systemic Tropheryma whipplei: Clinical presentation of 142 patients with infections diagnosed or confirmed in a reference center. Medicine (Baltimore).

[19] Meunier M, Puechal X, Hoppé E, Soubrier M, Dieudé P, Berthelot JM (2013). Rheumatic and musculoskeletal features of Whipple disease: A report of 29 cases. J. Rheumatol..

[20] Misbah SA, Ozols B, Franks A, Mapstone N (1997). Whipple’s disease without malabsorption: New atypical features. QJM.

[21] Günther U, Moos V, Offenmüller G, Oelkers G, Heise W, Moter A (2015). Gastrointestinal diagnosis of classical Whipple disease: Clinical, endoscopic, and histopathologic features in 191 patients. Medicine (Baltimore).

[22] Marth T (2015). Tropheryma whipplei, immunosuppression and Whipple’s disease: From a low-pathogenic, environmental infectious organism to a rare multifaceted inflammatory complex. Dig. Dis..

[23] Martinetti M, Biagi F, Badulli C, Feurle GE, Müller C, Moos V (2009). The HLA alleles DRB1*13 and DQB1*06 are associated to Whipple’s disease. Gastroenterology.

[24] Le Goff M, Cornec D, Guellec D, Marhadour T, Devauchelle-Pensec V, Jousse-Joulin S (2019). Peripheral-blood b-cell subset disturbances in inflammatory joint diseases induced by Tropheryma whipplei. PLoS ONE.

[25] Berent R, Auer J, Lassnig E, von Duvillard SP, Crouse SF, Tuppy H (2009). Whipple’s disease: Misdiagnosed as sarcoidosis with further tricuspid valve endocarditis and pulmonary embolism: A case report. BMJ Case Rep..

[26] de Henriques MS, da Paz AR, Gaertner ABP, Melo CIS, Filgueiras PL, Jerome RA (2016). Deep vein thrombosis as initial manifestation of Whipple disease. Case Rep. Gastroenterol..

[27] Moter A, Janneck M, Wolters M, Iking-Konert C, Wiessner A, Loddenkemper C (2019). Potential role for urine polymerase chain reaction in the diagnosis of Whipple’s disease. Clin. Infect. Dis..

[28] Tison A, Saraux A (2019). Potential role for urine polymerase chain reaction in the diagnosis of Whipple disease. Clin. Infect. Dis..

