# Publisher Correction: Rheumatological features of Whipple disease

**DOI:** 10.1038/s41598-021-94889-9

**Published:** 2021-08-04

**Authors:** Alice Tison, Pauline Preuss, Clémentine Leleu, François Robin, Adrien Le Pluart, Justine Vix, Guillaume Le Mélédo, Philippe Goupille, Elisabeth Gervais, Grégoire Cormier, Jean-David Albert, Aleth Perdriger, Béatrice Bouvard, Jean-Marie Berthelot, Nathan Foulquier, Alain Saraux

**Affiliations:** 1 UMR1227, Lymphocytes B et Autoimmunité, Université de Brest, INSERM, CHU Brest, LabEx IGO, Brest, France; 2 Rheumatology Unit, Centre National de Référence des Maladies Auto-Immunes Rares (CERAINO), CHU, Brest, France; 3 Rheumatology Unit, CHU, Nantes, France; 4 Rheumatology Unit, CHU, Angers, France; 5 Rennes, Service de Rhumatologie, Univ Rennes, INSERM, INRA, Institut NUMECAN (Nutrition Metabolisms and Cancer), 35000 Rennes, France; 6 Rheumatology Unit, CHU, Poitiers, France; 7 Rheumatology Department, University Hospital of Tours, EA 7501, GICC, University of Tours, Tours, France; 8 Rheumatology Unit, CHU, La Roche-sur-Yon, France; 9 Rheumatology Unit, Hôpital de la Cavale Blanche, BP 824, 29609 Brest cedex, France

Correction to: *Scientific Reports* https://doi.org/10.1038/s41598-021-91671-9, published online 10 June 2021

In the original version of this Article, the network of rheumatologists from the Grand Ouest “VICTOR HUGO” was incorrectly listed as an author in the author list.

Furthermore, the figure captions of Figures 1 and 2 in the original version of this Article contained a repeated error, where “Whipple disease” was incorrectly given as “Whipped disease”.

As a result, the caption of Figure 1,

“Proposed overactive immune actors involved in Whipped disease pathophysiology. *APC* antigen presentation cell, *DC* dendritic cell, *PNN* neutrophils polynuclears, *T* Lymphocytes T, *WD* Whipped disease.”

now reads:

“Proposed overactive immune actors involved in Whipple disease pathophysiology. *APC* antigen presentation cell, *DC* dendritic cell, *PNN* neutrophils polynuclears, *T* Lymphocytes T, *WD* Whipple disease.”

The caption of Figure 2,

“Proposed algorithm for Whipped disease in patients in whom rheumatism is suspected. *CSF* cerebro-spinal fluid, *CWD* classic Whipple disease, *PAS* periodic acid-Schiff, *SB* small bowel, *WD* Whipped disease. Cases of spondylodiscitis not considered in this algorithm. **Discuss CSF PCR in case of neurologic symptoms. *In case of chronic arthritis with negative synovial fluid PCR, discuss synovial biopsy. *NB* Data on cutaneous biopsy PCR were to scarce in our cohort integrate it on the algorithm.”

now reads:

“Proposed algorithm for Whipple disease in patients in whom rheumatism is suspected. *CSF* cerebro-spinal fluid, *CWD* classic Whipple disease, *PAS* periodic acid-Schiff, *SB* small bowel, *WD* Whipple disease. Cases of spondylodiscitis not considered in this algorithm. **Discuss CSF PCR in case of neurologic symptoms. *In case of chronic arthritis with negative synovial fluid PCR, discuss synovial biopsy. *NB* Data on cutaneous biopsy PCR were to scarce in our cohort integrate it on the algorithm.”

The errors have been corrected in the PDF and HTML versions of the Article.

