# Supplementary material for: Rheumatological features of Whipple disease
Source: Sci Rep. 2021 Jun 10;11:12278. doi: 10.1038/s41598-021-91671-9 (PMC8192552; doi:10.1038/s41598-021-91671-9)
Supplement: Supplementary file 1 — Supplementary Tables. [file 41598_2021_91671_MOESM1_ESM.docx]

**Supplementary Table 1 – Keywords used in BIBOT article preselection for systematic literature review**

| *Tropheryma whipplei*  **Clinic**  **Classical- Classic Whipple’s disease**  **Immunohistochemistry**  **Gold**  **Microscopy**  **Duodenum**  **Histology**  **Macrophage**  **PAS**  **Digestive**  **Localized**  **Probable**  **Rheumatic**  **Rheumatology**  **Joint**  **Arthritis**  **Arthralgia**  **Spondyloarthritis**  **Spondylarthritis**  **Axial**  **Spondylitis**  **Sacroiliitis** |
| --- |

**Supplementary Table 2– Series comparing clinical and biological features of CWD and rheumatological LWD selected from the systematic literature review**

| Series | Year | Period of recruitment | Whipple disease  (n) | CWD  (n) | LWD  (n) | LW arthritis  (n) |
| --- | --- | --- | --- | --- | --- | --- |
| Hujoel et al (16) | 2019 | 1995-2010 | 33 | 18 | 15 | 4 |
| Crews and al (15) | 2018 | 1994-2016 | 33 | 18 | 15 | 9 |
| Lehman et al (14) | 2017 | 2006-2014 | 18 | 7 | 11 | 3 |
| Glaser et al (17) * | 2017 | 2010-2015 | 7 | 3 | 4 | 4 |
| Meunier et al (19) * | 2013 | 1977-2011 | 28 | 18 | 3 ** | 2 |
| Lagier et al (18) | 2010 | 2000-2010 | 142 | 113 | 29 | 2 |
| Fenollar et al (10) | 2008 | 2003-2007 | 71 | 55 | 16 | 1 |
| Misbah et al (20) | 1997 | ND | 5 | 0 | 5 | 1 |

***Legend:*** *CWD: Classic Whipple disease LWD: Localized Whipple disease*

******* *Rheumatological series*

*** + 7 patients with equivalent “Arthropathic Whipple disease” with negative PAS staining, negative CSF and synovial fluid PCR (diagnoses made with small-bowel, saliva, stool, skin and/or blood sample PCR)*

**Supplementary Table 3 – Whipple disease diagnosis according to teaching hospital centre**

| Centres | CWD  N=11 | NCWD | | | Total  N=68 |
| --- | --- | --- | --- | --- | --- |
|  |  | LWD  N=24 | AWD  N=15 | PAWD  N=18 |  |
| Angers | 0 | 1 | 0 | 7 | 8 |
| Brest | 2 | 7 | 5 | 2 | 16 |
| La Roche-sur-Yon | 2 | 2 | 1 | 2 | 7 |
| Nantes | 5 | 3 | 6 | 6 | 20 |
| Poitiers | 2 | 2 | 1 | 1 | 6 |
| Rennes | 0 | 7 | 1 | 0 | 8 |
| Tours | 0 | 2 | 1 | 0 | 3 |

*Legend: AWD = Arthropathic Whipple disease; CWD = Classic Whipple disease; LWD = Localized Whipple disease; NCWD = Nonclassic Whipple disease; PAWD = Probably Arthropathic Whipple disease*

**Supplementary Table 4 – Joints affected by arthritis and tenosynovitis in the Whipple disease cohort**

| Joint arthritis (n) % | Whipple disease patients  n=68 |
| --- | --- |
| Knee | 34 (50) |
| Ankle | 33 (49) |
| Wrist | 29 (43) |
| Metacarpophalangeal joint | 13 (19) |
| Proximal interphalangeal joint | 9 (13) |
| Wrist tenosynovitis | 9 (13) |
| Shoulder/subacromial bursitis | 7 (10) |
| Elbow/hygroma | 7 (10) |
| Midfoot | 5 (7.4) |
| Ankle/foot tenosynovitis | 4 (5.9) |
| Hip | 3 (4.4) |
| Dactylitis | 3 (4.4) |
| Acromioclavicular joint | 1 (1.5) |

**Supplementary Table 5 – Evolution after antibiotic treatment according to Whipple disease entity**

|  | CWD  N=11 | LWD  N=24 | AWD  N=15 | PAWD  N=18 | Total  N=68 |
| --- | --- | --- | --- | --- | --- |
| IRIS | 2/11 (18) | 1/20 (5) | 2/15 (13) | 0/18 (0) | 5/64 (7.8) |
| Negative PCR at antibiotic discontinuation | 9/11 (82) | 11/13 (85) | 8/8 (100) | 8/11 (73) | 36/43 (84) |
| Relapse | 5/11 (45) | 7/22 (32) | 6/15 (40) | 3/17 (18) | 21/65 (32) |
| Evolution towards chronic rheumatism | 2/11 (18) | 1/22 (4.5) | 2/14 (14) | 2/17 (12) | 7/64 (11) |

*Legend: AWD = Arthropathic Whipple disease; CWD = Classic Whipple disease; IRIS= Immune reconstitution inflammatory syndrome; LWD = Localized Whipple disease; PAWD = Probably Arthropathic Whipple disease*

**Supplementary Table 6 – Characteristics of Whipple disease patients whose conditions evolved towards chronic rheumatism**

|  | PAS staining at diagnosis | Positive TW PCR at diagnosis | PCR after antibiotherapy | Final diagnosis | Treatment |
| --- | --- | --- | --- | --- | --- |
| Patient 1 | - | Joint fluid | Negative | CUR after WD | Methotrexate + etanercept |
| Patient 2 | + | Stool, saliva, duodenum, blood | Still positive on duodenum, PAS + | Spondyloarthritis after WD | Punctual NSAID |
| Patient 3 | - | Stool, saliva, duodenum | Negative | Psoriatic arthritis + WD * | Methotrexate + doxycycline + low-dose GC |
| Patient 4 | + | Duodenum, saliva | Still positive | CUR | NSAID |
| Patient 5 | UR | Stool, saliva | Still positive | CUR + psoriasis | Secukinumab |
| Patient 6 | - | Stool, saliva, duodenum, blood | Negative in stool, saliva, joint fluid | CUR | Intra-articular infiltrations |
| Patient 7 | UR | Stool, saliva, duodenum | Negative | Spondyloarthritis + Crohn’s disease + WD | Doxycycline + hydroxychloroquine + adalimumab |

*Legend: CUR: chronic unclassified rheumatism; GC: Glucocorticoids; UR: Unrealized; NSAID: Non-steroidal anti-inflammatory drug; PAS: Periodic acid-Schiff; TW: Tropheryma whipplei; WD: Whipple disease*

**+Anti-CCP antibody occurrence*
