# Supplementary figures and images for: Rheumatological features of Whipple disease
Source: Sci Rep. 2021 Jun 10;11:12278. doi: 10.1038/s41598-021-91671-9 (PMC8192552; doi:10.1038/s41598-021-91671-9)

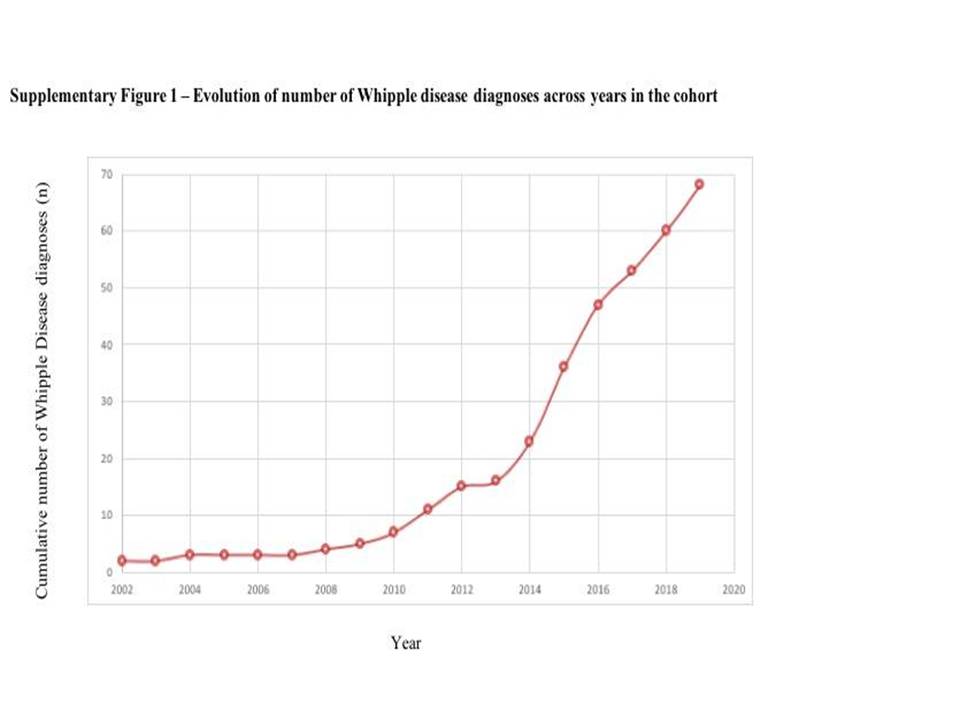

Supplement: Supplementary file 2 — Supplementary Figure. [file 41598_2021_91671_MOESM2_ESM.jpg]
